# RPSA enhances African swine fever virus entry via caveola-mediated endocytic pathway

**DOI:** 10.1128/jvi.00131-26

**Published:** 2026-05-11

**Authors:** Chuanxia Liu, Tingting Li, Yibing Wang, Fei Zhao, Jiangnan Li, Li Huang, Changjiang Weng

**Affiliations:** 1 Professional Laboratory for African Swine Fever (Harbin), Harbin Veterinary Research Institute, Chinese Academy of Agricultural Sciences (CAAS) 111613 , Harbin, China; 2 Heilongjiang Provincial Key Laboratory of Veterinary Immunology 111613 , Harbin, China; The Ohio State University, Columbus, Ohio, USA

**Keywords:** African swine fever virus, E120R, RPSA, lipid raft, caveolin-1

## Abstract

African swine fever virus (ASFV) poses a severe threat to the global pig industry, yet the precise mechanisms of its entry into host cells are not fully understood. In this study, we identified the host ribosomal protein SA (RPSA), a protein located in lipid rafts, as a critical viral receptor that facilitates ASFV entry. Immunoprecipitation-mass spectrometry (IP-MS) screening results identified RPSA as a binding partner of the ASFV capsid protein pE120R, which was further confirmed by co-immunoprecipitation (Co-IP) and immunofluorescence assays (IFA) in ASFV-infected cells. Moreover, knockdown of RPSA expression in porcine alveolar macrophages (PAMs) by siRNA significantly reduced ASFV attachment and internalization. In contrast, ectopic overexpression of RPSA in permissive CV-1 cells enhanced the attachment and internalization of ASFV virions. Additionally, we observed that both pharmacological inhibition of caveola-mediated endocytosis (CavME) with nystatin and genetic knockdown of caveolin-1 (CAV1) decreased caveola-mediated ASFV virions endocytosis and internalization. Mechanistically, RPSA interacts with CAV1, a major structural protein of caveolae. Domain mapping showed that pE120R mainly binds to the LR2 and LR3 domains of RPSA, while RPSA interacts with CAV1 via its 16B domain, forming a pE120R-RPSA-CAV1 complex that facilitates ASFV entry. Overall, these results demonstrate that RPSA works as a potential host receptor linking ASFV pE120R to the CAV1-mediated endocytic pathway, facilitating viral entry through caveola-mediated endocytosis. These findings advance our understanding of ASFV entry mechanism and offer a potential target for antiviral strategies against ASFV.

**IMPORTANCE:**

African swine fever threatens the global pig industry, yet its entry route remains unclear. Here, host ribosomal protein SA (RPSA) was identified as a binding partner of ASFV capsid protein pE120R. Knockdown of RPSA expression using siRNA in porcine alveolar macrophages reduced ASFV attachment and internalization, whereas ectopic overexpression in CV-1 cells enhanced the uptake of ASFV virions. Pharmacological blockade of caveola-mediated endocytosis (CavME) with nystatin or genetic silencing of caveolin-1 (CAV1) similarly impaired infection, indicating that ASFV entry into its target cells is CavME dependent. Domain mapping showed that pE120R binds LR2 and LR3 of RPSA, while RPSA uses its 16B domain to recruit CAV1, assembling a pE120R-RPSA-CAV1 entry complex. Thus, RPSA bridges ASFV to the CAV1-driven caveolar pathway. These findings define a novel receptor-mediated entry mechanism for ASFV virions and reveal that RPSA-CAV1 pathway may be an actionable antiviral target.

## INTRODUCTION

African swine fever (ASF) poses a threat to the global pig industry, leading to significant economic losses (1, 2). African swine fever virus (ASFV), first identified in Kenya in 1921, is a highly contagious and often deadly virus that affects both domestic and wild pigs, with mortality rates reaching up to 100%. At present, there are no effective vaccines or antiviral treatments available for ASFV. Control efforts mainly depend on biosecurity measures, including strict quarantine and the culling of infected animals (2). Gaining a better understanding of the epidemiology and pathogenesis is crucial for developing effective prevention strategies and reducing its impact on the swine industry (2).

ASFV, the only member of the *Asfaviridae* family, is a double-stranded DNA virus (3). ASFV genome ranges from 170 to 190 kilobase pairs (kbp) and encodes approximately 150 to 200 viral proteins (4). ASFV virions exhibit an icosahedral morphology with an icosahedral multilayered structure, arranged from the innermost to the outermost layer: the nucleoid, core-shell, inner lipid envelope, and capsid from the inner to the outer layers (3, 5). Mature ASFV acquires an additional external lipid envelope during budding through the host cell plasma membrane. Previous studies have shown that this external lipid envelope of ASFV virion is not essential for ASFV infectivity, implying that the entry of ASFV into the target cells may primarily depend on the function of viral capsid proteins present on the surface of ASFV virions (3, 4). Among several capsid proteins of ASFV, pE120R is relatively conserved, and several monoclonal antibodies against pE120R have exhibited neutralizing activity (6, 7), suggesting a potential role for pE120R in mediating ASFV entry.

Previous studies have identified several host factors that influence the entry of ASFV, including CD163 (8), CD1d (9), and AXL (10). However, experiments involving the knockdown or knockout of these host factors have demonstrated that ASFV is still capable of infecting these cells (8–10). This observation suggests that additional co-receptors or alternative endocytic pathways may be necessary for ASFV entry.

Lipid rafts are small, dynamic microdomains within the plasma membrane where cholesterol and sphingolipids are enriched. Lipid rafts are not only present in the plasma membrane but are also found in intracellular membranes and extracellular vesicles (11). A growing body of evidence indicates that lipid rafts are involved in viral attachment and recruitment, as well as in mediating both endocytic and non-endocytic pathways utilized by certain viruses to enter host cells (11). The process of viral entry into host cells is a complex process and initially requires the binding of the virus to the cell surface through receptor or co-receptors (12). Lipid rafts serve to concentrate viral receptors or co-receptors within the cell membrane, thereby enhancing the probability of virus-receptor interactions. Some cellular receptors and co-receptors are constitutively expressed in rafts, such as angiotensin-converting enzyme 2 (ACE2), the receptor for severe acute respiratory syndrome coronavirus (SARS-CoV) and SARS-CoV-2 (13). The relocation of receptors and co-receptors into rafts has been documented during infections by human herpesvirus type 6 (HHV-6) (14), Kaposi’s sarcoma-associated herpesvirus (KSHV) (15), vaccinia virus (16), HIV-1 (17), and enterovirus D68 (EV-D68) (18). Collectively, these findings underscore the pivotal role of lipid rafts in facilitating viral entry into host cells (11, 19).

Ribosomal protein SA (RPSA), also known by several alternative names, including the 37 kDa laminin receptor precursor protein (37LRP), the 67 kDa laminin receptor (67LR), the 32 kDa laminin binding protein (LBP), and p40, performs a diverse range of biological functions (20). RPSA is predominantly localized within multiple cellular compartments, including the nucleus, cytoplasm, cell membrane, and extracellular vesicles (21–23). Notably, RPSA is primarily situated in the lipid rafts of the cell surface (24). In the context of microbial infections, RPSA has been implicated in the pathogenesis of meningitis caused by different pathogens, such as *Streptococcus pneumoniae*, *Neisseria meningitidis*, *Haemophilus influenzae*, and *Escherichia coli* K1 (25–27). Furthermore, RPSA also functions as a critical host cell receptor facilitating the entry of prions (28), dengue virus (29), and adeno-associated virus (30).

In this study, we identified RPSA, a lipid raft-associated protein, as a novel interacting partner of ASFV pE120R by conducting co-immunoprecipitation coupled with mass spectrometry (Co-IP/MS) analysis using an antibody against ASFV capsid pE120R. Subsequent experiments demonstrated that knockdown of RPSA expression in PAMs significantly inhibited ASFV entry, whereas overexpression of RPSA significantly increased ASFV entry. Furthermore, our findings demonstrate that the RPSA-mediated assembly of a pE120R-RPSA-CAV1 complex facilitates ASFV entry via the caveola/lipid raft-mediated endocytosis. Collectively, our findings advance our understanding of the molecular mechanisms underlying ASFV entry into the permissive cells.

## RESULTS

### The host protein RPSA interacts with ASFV pE120R

**Fig 1:**
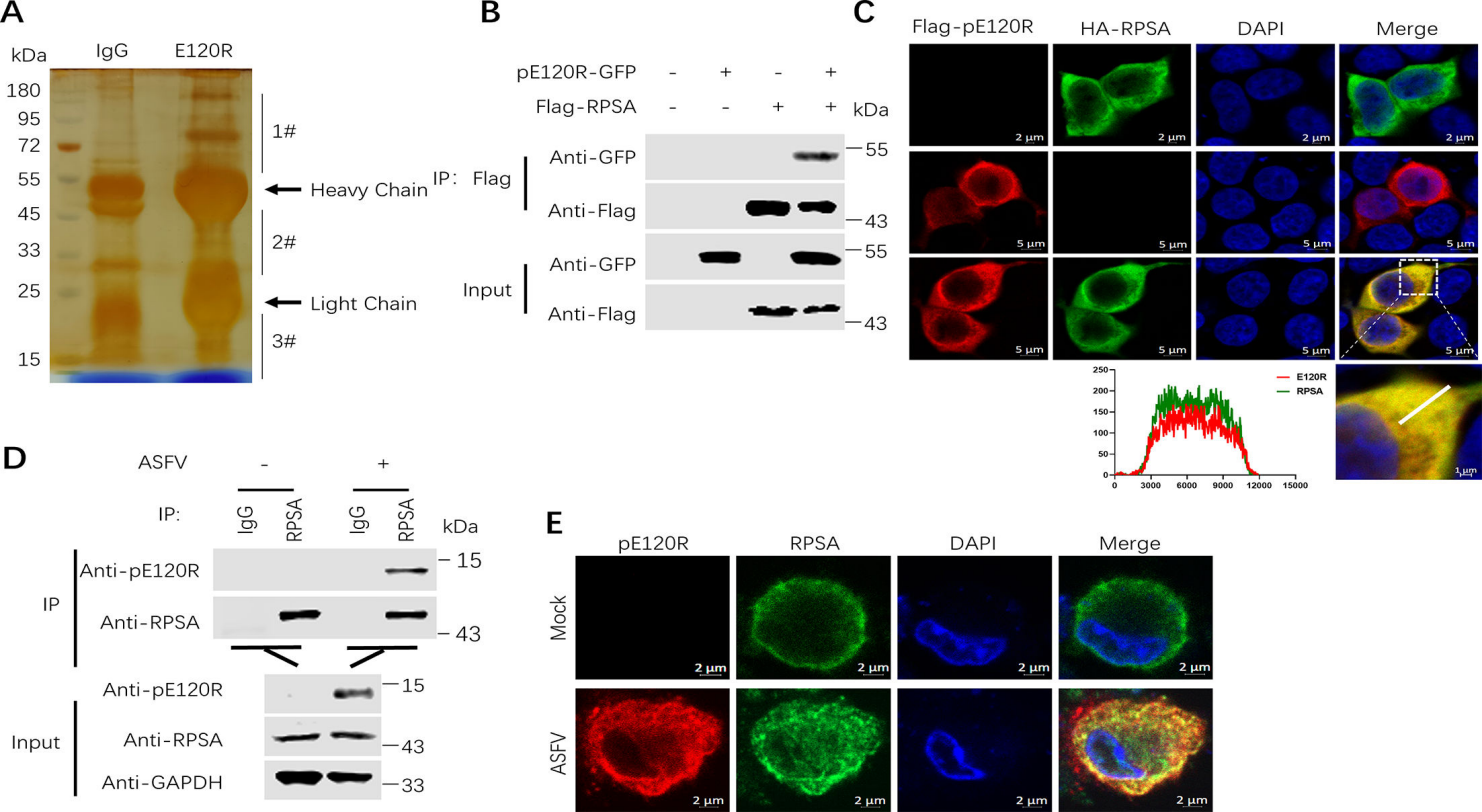
Host protein RPSA was identified as a novel binding partner of ASFV capsid protein pE120R. (**A**) Co-IP combined with silver staining was employed to assess the expression of pE120R in ASFV-infected PAMs and to identify proteins that interact with pE120R. PAMs were either mock-infected or infected with ASFV-HLJ/18 at a multiplicity of infection (MOI) of 1 for 24 h. The cell lysates were incubated with an anti-pE120R antibody or mouse IgG control for 8 h at 4°C. ASFV pE120R-binding proteins were eluted from protein A + G Sepharose and subjected to SDS-PAGE, followed by silver staining. The pE120R bands labeled in 1#, 2#, and 3# were excised for mass spectrometry analysis. (**B**) The cell lysates from HEK293T cells co-transfected with plasmids expressing GFP-pE120R and Flag-tagged RPSA were subjected to Co-IP with an anti-Flag antibody. Both the immunoprecipitates and whole-cell lysates (input) were analyzed by immunoblotting with anti-Flag and anti-GFP antibodies, respectively. (**C**) HEK293T cells were co-transfected with plasmids expressing Flag-pE120R and HA-RPSA. pE120R (red) and RPSA (green) were detected by immunofluorescence staining with anti-Flag or anti-HA antibodies, respectively. Nuclear staining was performed with DAPI. The degree of co-localization between pE120R and RPSA was analyzed using the Pearson correlation coefficient. Scale bar = 5 µm. (**D**) PAMs were either mock-infected or infected with ASFV HLJ/18 at MOI of 5, and cell lysates were analyzed to detect the interaction between the interaction of endogenous RPSA and pE120R. Co-IP was performed using antibodies against pE120R, RPSA, or IgG control, followed by protein A + G Sepharose precipitation. (**E**) PAMs were mock-infected or infected with ASFV HLJ/18 at an MOI of 5, and the localization of endogenous RPSA and pE120R in PAM was detected by IFA with anti-pE120R and anti-RPSA antibodies, respectively. Nuclear staining was conducted with DAPI. Co-localization analysis was performed using the Pearson correlation coefficient. Scale bar = 2 µm.

As a component of the viral capsid, the role of ASFV pE120R in mediating viral entry into host cells remains inadequately characterized. To elucidate its role, we performed a Co-IP/MS assay using an antibody against pE120R to identify its principal host binding partners (Fig. 1A). Mass spectrometric results revealed numerous potential pE120R-interacting proteins were identified (Table S1). Protein “scores” represent the cumulative confidence based on peptide identification, with higher scores indicating greater reliability. Notably, RPSA emerged as a high-confidence binding partner of pE120R. Validation of the interaction between RPSA and pE120R was confirmed by Co-IP and indirect fluorescent assay (IFA) (Fig. 1B and C). Furthermore, endogenous RPSA was also found to interact and colocalize with ASFV pE120R in PAMs infected with ASFV-WT for 24 h (Fig. 1D and E). Collectively, these data substantiate the interaction between pE120R and RPSA.

### RPSA is critical for ASFV infection

**Fig 2:**
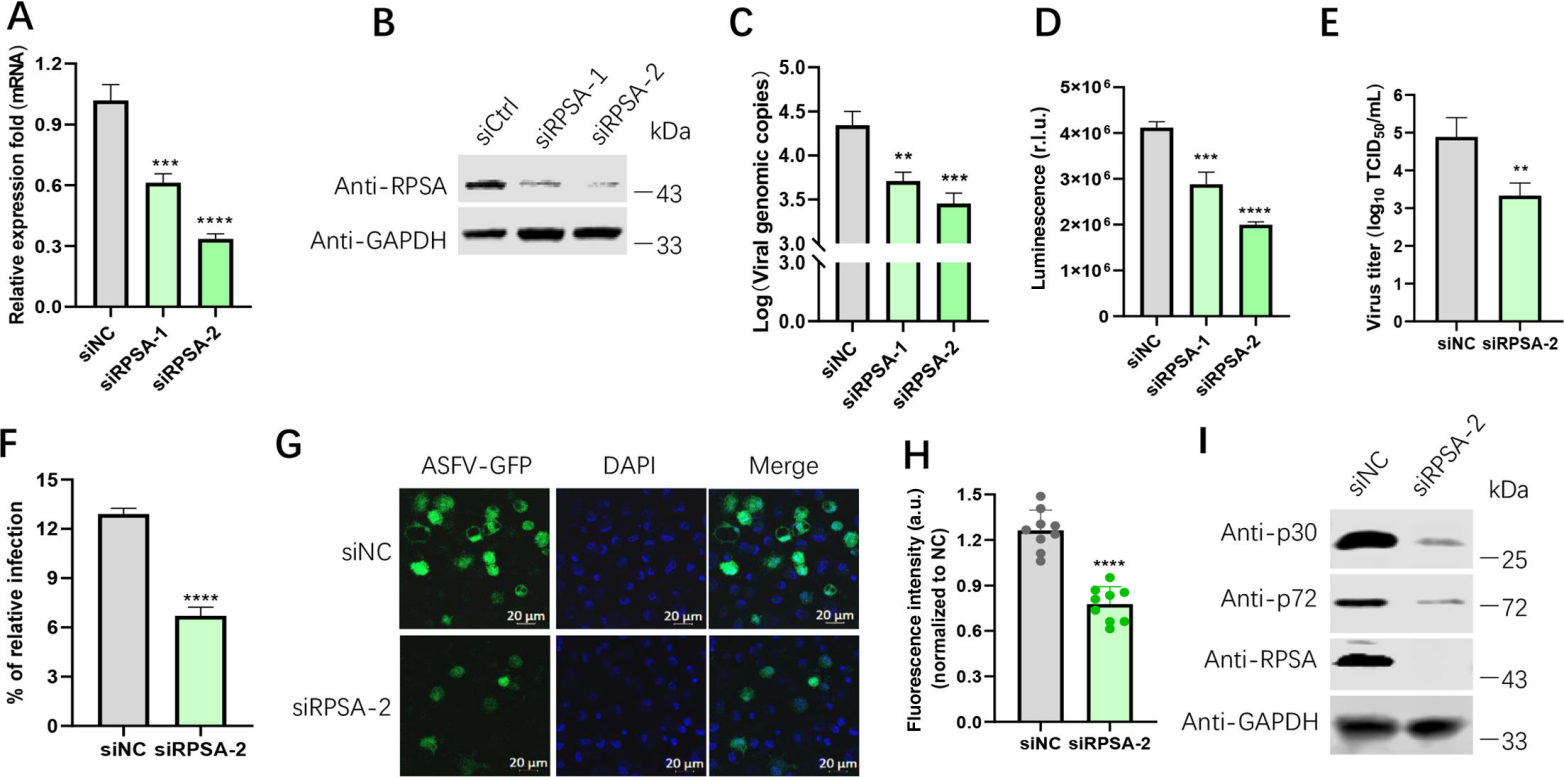
RPSA functions as a critical host factor in ASFV infection. (**A–I**) PAMs were transfected for 24 h with either a scrambled siRNA control (siNC) or siRNA targeting RPSA. Subsequently, cells were infected with recombinant ASFV expressing Gaussia luciferase and GFP (rASFV-Gluc-GFP) at an MOI of 1 and incubated at 37°C for 24 h. RPSA mRNA expression was quantified by qPCR (**A**), and RPSA protein levels were assessed via western blot analysis (**B**). ASFV genomic DNA within the cells was measured by qPCR (**C**), while viral replication was further evaluated by luciferase activity assays (**D**). Viral titers in the culture supernatants were determined using the TCID_50_ assay (**E**). The proportion of infected cells was analyzed through flow cytometry (**F**) and immunofluorescence assays (IFA) (**G and H**). Additionally, the expression levels of viral proteins p30 and p72 were examined by western blotting (**I**). Data are presented as mean ± standard deviation from three independent experiments. Statistical significance is indicated as follows: ****, *P* < 0.0001; ***, *P* < 0.001; **, *P* < 0.01.

**Fig 3:**
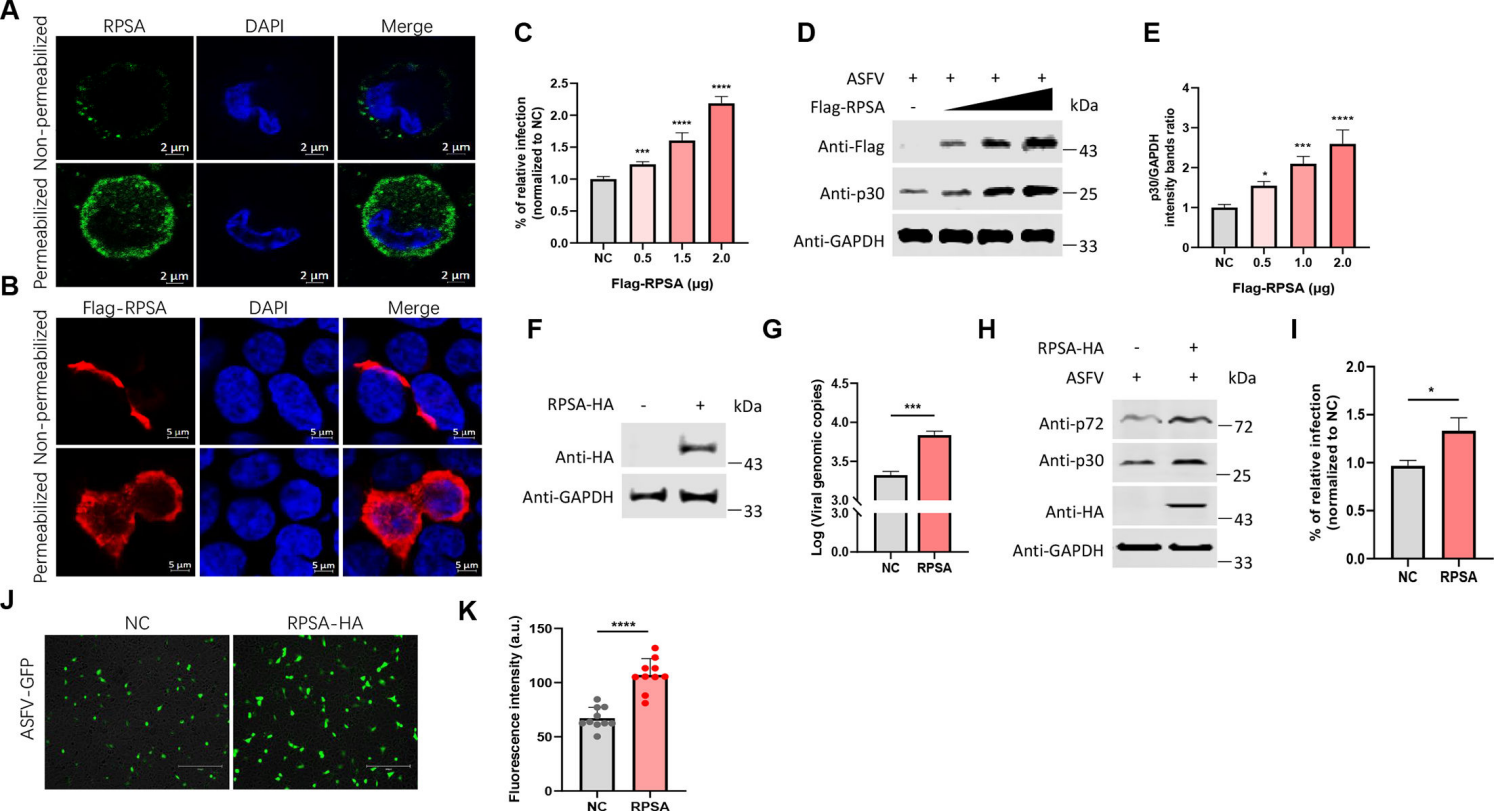
Ectopic expression of RPSA promotes ASFV infection in CV-1 cells. (**A**) PAMs were fixed with 4% paraformaldehyde, either in the presence or absence of Triton X-100, followed by immunostaining using anti-RPSA antibodies. Cell nuclei were counterstained with DAPI (blue). Scale bar = 2 µm. (**B**) HEK293T cells overexpressing Flag-tagged RPSA were similarly fixed with 4% paraformaldehyde, with or without Triton X-100 treatment, and immunostained using anti-Flag antibodies. Nuclei were stained with DAPI (blue). Scale bar = 5 µm. (**C–E**) CV-1 cells expressing RPSA were infected with recombinant ASFV expressing Gaussia luciferase and GFP (rASFV-Gluc-GFP) at an MOI of 1 and incubated at 37°C for 24 h. The proportion of infected cells was quantified via flow cytometry (**C**). Expression levels of viral proteins p30 and p72 were assessed by western blot analysis (**D**), and relative protein quantities were determined using ImageJ software (**E**). Data are presented as mean ± standard deviation from three independent experiments. (**F**) Generation of CV-1 cells stably expressing RPSA. CV-1 cells were transduced with a lentiviral virus expressing RPSA. The levels of RPSA protein were assessed by western blot with an anti-RPSA antibody. GAPDH was used as an internal control. (**G–K**) CV-1 cells were infected with recombinant ASFV (rASFV-Gluc-GFP) at 1 MOI and incubated at 37°C for 24 h. ASFV genomic DNA levels within the cells were quantified by qPCR (**G**). The abundance of viral proteins p30 and p72 was assessed via western blotting (**H**). The proportion of infected cells was determined through flow cytometry (**I**) and IFA (**J and K**). Data are presented as the mean ± standard deviation from three independent experiments. Statistical significance is indicated as follows: ****, *P* < 0.0001; ***, *P* < 0.001; *, *P* < 0.05.

To evaluate the role of RPSA in ASFV infection, knockdown of RPSA expression in PAMs was performed by transfection with siRNA targeting RPSA (siRPSA) (Fig. 2A and B), and subsequently ASFV replication was assessed. Our findings revealed that the genomic DNA levels (Fig. 2C), luciferase reporter activity (Fig. 2D), viral titer (Fig. 2E), and percentage of infected cells (Fig. 2F through H) were significantly decreased in PAMs treated with siRPSA compared to the control siRNA (siNC). Similarly, we noticed that ASFV p30, a viral early expression protein, was significantly reduced following the knockdown of RPSA expression (Fig. 2I). To further elucidate the impact of ectopic expression of RPSA on ASFV infection, we first examined subcellular localization of RPSA, observing that there is significant enrichment of RPSA on the plasma membrane alongside cytoplasmic distribution in PAMs (Fig. 3A and B). Furthermore, overexpression of RPSA in CV-1 cells enhanced ASFV infection in a dose-dependent manner (Fig. 3C), accompanied by an increase in ASFV protein levels (Fig. 3D and E). Consistent with these results, we found that viral infection rate increased in the presence of RPSA-overexpressed cells (Fig. 3F through K). Taken together, RPSA is associated with ASFV infection.

### RPSA contributes to the attachment of ASFV virions

**Fig 4:**
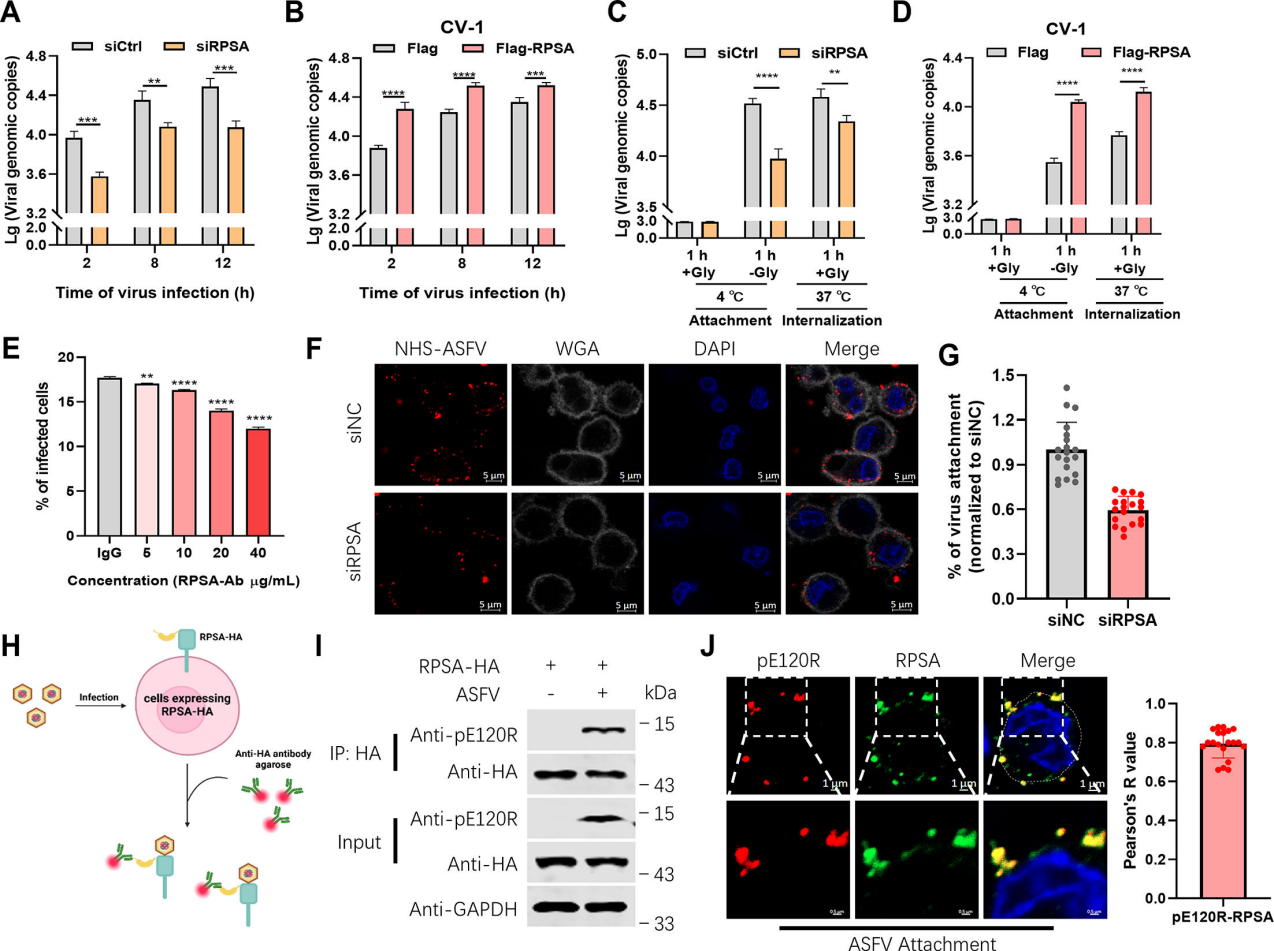
The critical role of RPSA in ASFV attachment. (**A and B**) Detection of RPSA expression in PAMs treated with siRNA or in CV-1 cells overexpressing RPSA. Subsequently, these cells were infected with ASFV HLJ/2018 at a multiplicity of infection (MOI) of 2. Viral infection rates were quantified by qPCR at 2, 8, and 12 hpi. (**C and D**) Knockdown of RPSA expression in PAMs and overexpressing RPSA in CV-1 cells. Subsequently, the cells were infected at 4°C with ASFV HLJ/2018 at an MOI of 2. After 1 h, cells were washed and harvested for viral genome quantification via qPCR. Additional samples were then incubated at 37°C for another 1 h, followed by washing and collection for viral genome analysis. Incubation at 4°C in the presence of glycine (+Gly) was employed to remove unbound virus particles from the cell surface. (**E**) PAMs were incubated with anti-RPSA antibody first at the indicated concentrations for 1 h at 4°C, followed by infection with rASFV-Gluc-GFP at an MOI of 1. IgG as a negative control. At 24 hpi, infection rates were assessed at 24 hpi by flow cytometry analysis. (**F and G**) Statistical analysis of NHS-ASFV attachment. To quantify viral binding, cell membranes were labeled with wheat germ agglutinin (WGA, gray). ASFV virions colocalized with the cell membrane (red) were considered bound, whereas internalized ASFV virions (red) were distinguished accordingly. Over 200 cells per sample were analyzed to calculate the percentage of cells positive for internalized NHS-ASFV. Scale bar = 5 µm. (**H**) A schematic representation of the methodology used to detect interactions between ASFV and RPSA during viral infection. (**I**) Co-IP and immunoblotting confirmed the interaction of RPSA with ASFV. (**J**) PAMs were infected with ASFV at an MOI of 100 for 1 h at 4°C, followed by fixation. Non-permeabilized cells were immunostained for the viral protein pE120R, and nuclei were counterstained with DAPI (blue). Co-localization of RPSA (green) with pE120R (red) was observed. Scale bar = 1 µm. All data are presented as mean ± standard deviation derived from three independent experiments. ****, *P* < 0.0001; ***, *P* < 0.001; **, *P* < 0.01.

ASFV virions are known to initially bind to the cell membrane before being internalized into targeting cells, which eventually leads to the release of viral genomes into the cytoplasm for viral replication (3). To investigate which stage of ASFV replication can be affected by RPSA, we first qualified the viral genomic copies in PAMs with RPSA knockdown and in CV-1 cells overexpressing RPSA at 2, 8, and 12 h post-infection (hpi). As shown in Fig. 4A and B, knockdown of RPSA expression resulted in a reduction of the viral genomic copies, whereas overexpression of RPSA led to an increase in ASFV infection from 2 hpi onward, suggesting a critical role for RPSA in ASFV entry. To further validate these results, we assessed the impact of RPSA on viral adhesion and internalization. As shown in Fig. 4C, RPSA knockdown reduced the number of virions attached to the cell surface, consequently reducing ASFV internalization. Conversely, RPSA overexpression in CV1 cells enhanced both adhesion and internalization of ASFV virions (Fig. 4D). Additionally, treatment with an anti-RPSA antibody inhibited ASFV infection in a dose-dependent manner (Fig. 4E). To further confirm RPSA’s involvement in viral attachment, confocal microscopy was used to visualize the attachment of NHS-labeled ASFV virions. As shown in Fig. 4F and G, a significant decrease in viral attachment was observed in PAMs transfected with siRPSA compared to siNC. Furthermore, immunoprecipitation assays revealed a specific interaction between RPSA proteins and ASFV particles during viral attachment, as evidenced by the precipitation of viral particles by RPSA, whereas no enrichment of the ASFV protein pE120R was detected in the control samples (Fig. 4H and I). High-resolution immunofluorescence imaging further confirmed the colocalization of RPSA and pE120R during ASFV attachment (Fig. 4J). Collectively, these data indicate that ASFV virions attach to RPSA on the cell membrane during the initial stages of ASFV infection.

### RPSA participates in internalization of ASFV virions

**Fig 5:**
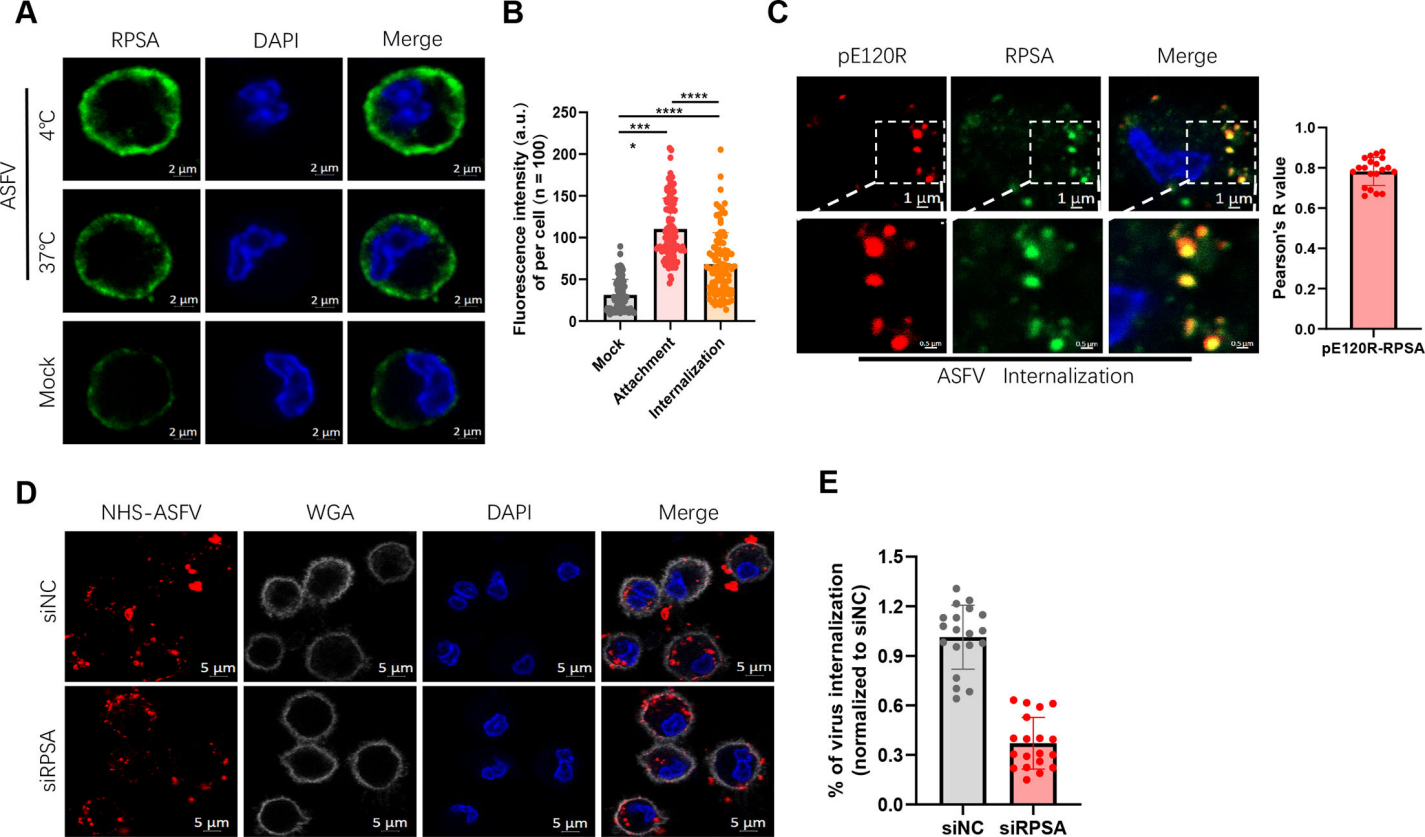
RPSA is involved in ASFV internalization. (**A and B**) PAMs were either mock-infected or infected with ASFV-WT at 4°C or 37°C for 1 h, followed by fixation. Non-permeabilized cells were immunostained for RPSA (green), and nuclei were counterstained with DAPI (blue). Scale bar = 2 µm (**A**). Fluorescence intensity was quantified using confocal laser scanning microscopy (**B**). (**C**) PAMs were infected with ASFV at a MOI of 100 at 37°C for 1 h, then fixed. Following permeabilization, cells were immunostained for the viral protein pE120R, with nuclei stained by DAPI (blue). Scale bar = 1 µm. Co-localization between RPSA and pE120R was observed. (**D and E**) Quantitative analysis of NHS-labeled ASFV internalization was performed. To assess internalization, wheat germ agglutinin (WGA)-labeled cell membranes (gray) served as a reference; virus particles co-localizing with the membrane (red) were classified as bound, whereas those located internally (red) were considered internalized. Over 200 cells per sample were analyzed, and the percentage of cells positive for internalized NHS-ASFV was calculated. Scale bar = 5 µm. Data are presented as mean ± standard deviation from three independent experiments. Statistical significance was denoted as ****, *P* < 0.0001.

Confocal microscopy results revealed a reduction in the fluorescence intensity of RPSA on the cell membrane surface during the internalization of ASFV virions (Fig. 5A and B), implying that RPSA may be internalized concomitantly with the ASFV virions. To further investigate this, laser confocal microscopy was employed to examine the co-localization of RPSA and pE120R during ASFV virions internalization. We found a substantial co-localization between RPSA and internalized viral particles, with a Pearson correlation coefficient of 0.88 (Fig. 5C). Moreover, knockdown of RPSA expression in PAMs reduced the NHS-labeled ASFV particles internalization (Fig. 5D and E). Collectively, these findings suggest that RPSA plays a contributory role in the internalization process of ASFV.

### RPSA participates in caveola-mediated endocytosis during viral infection

**Fig 6:**
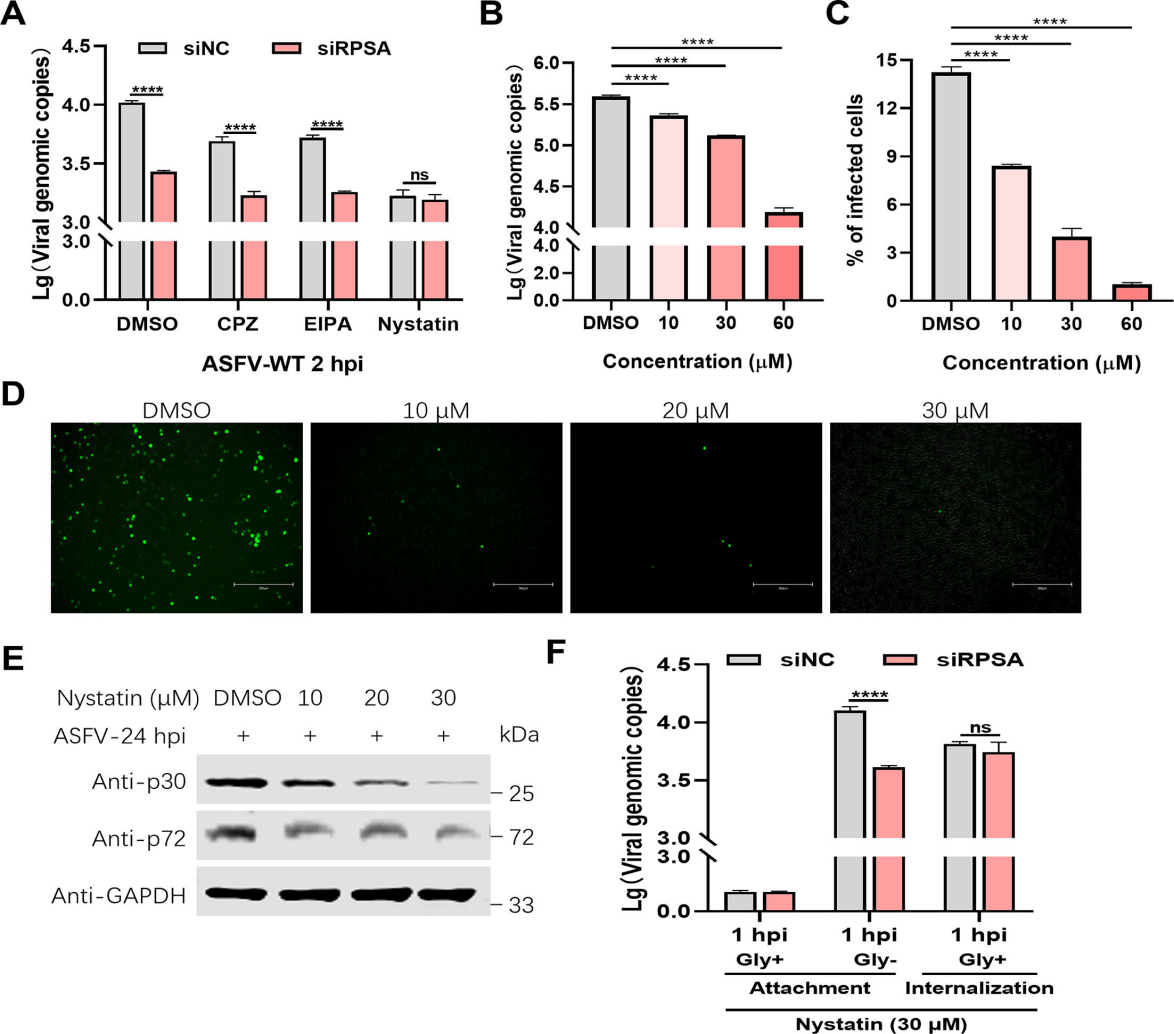
ASFV enters PAMs via the caveola-mediated endocytosis pathway. (**A**) PAMs were transfected with either scrambled siRNA control (siNC) or two distinct siRNAs targeting RPSA for 24 h. Subsequently, the cells were pretreated with chlorpromazine (CPZ, 30 μM), 5-(N-ethyl-N-isopropyl) amiloride (EIPA, 50 μM), or nystatin (30 μM) for 30 min, followed by infection with ASFV-WT at 0.5 MOI for 1 h at 37°C. Viral internalization was quantified via qPCR. Dimethyl sulfoxide (DMSO) served as the negative control. (**B–E**) PAMs were pretreated with nystatin for 30 min prior to infection with rASFV-GFP at an MOI of 0.5 for 24 h at 37°C. ASFV genomic DNA levels within cells were assessed by qPCR (**B**). The proportion of infected cells was determined through flow cytometry (**C**) and IFA (**D**). Expression levels of viral proteins p30 and p72 were evaluated by western blot analysis (**E**). DMSO was used as the negative control. (**F**) PAMs were pretreated with nystatin (30 μM) for 30 min and then infected with ASFV-WT (MOI=0.5) for 1 h at 4°C to allow virus binding. Following incubation, cells were washed to remove unbound virus and subsequently incubated at 37°C for 1 h to permit internalization. Uninternalized viral particles were removed by washing with 0.2 M glycine (pH 3.0). Internalized virus was quantified by qPCR. Data are presented as mean ± standard deviation from three independent experiments. Statistical significance is indicated as follows: ****, *P* < 0.0001; ns, not significant (*P* > 0.05).

Endocytic pathways have been extensively characterized in viral entry, including clathrin-mediated endocytosis (CME), caveola/lipid raft-mediated endocytosis (CavME), and macropinocytosis. Specific inhibitors, such as chlorpromazine (CPZ) for CME, 5-(N-ethyl-N-isopropyl) amiloride (EIPA) for macropinocytosis, and nystatin for CavME, are commonly employed to dissect these pathways. To investigate which pathway RPSA is involved in during virions internalization in ASFV infection, PAMs were transfected with either siRPSA or siNC, followed by treatment with each inhibitor prior to ASFV infection. As shown in Fig. 6A, knockdown of RPSA expression significantly attenuated the inhibitory effect of nystatin on viral entry, whereas the effects of CPZ and EIPA remained unaffected. This finding suggests a specific role for RPSA-mediated endocytosis of ASFV virions via the CavME pathway.

To further substantiate ASFV internalization via CavME, cells were pretreated with nystatin before viral exposure. Our results demonstrated that nystatin inhibited viral infection in a dose-dependent manner (Fig. 6B through E). Notably, RPSA depletion abrogated nystatin’s capacity to inhibit viral internalization (Fig. 6F), suggesting that RPSA played a key role in this process. Collectively, these findings indicate that RPSA contributes to caveola-mediated endocytosis process during ASFV entry.

**Fig 7:**
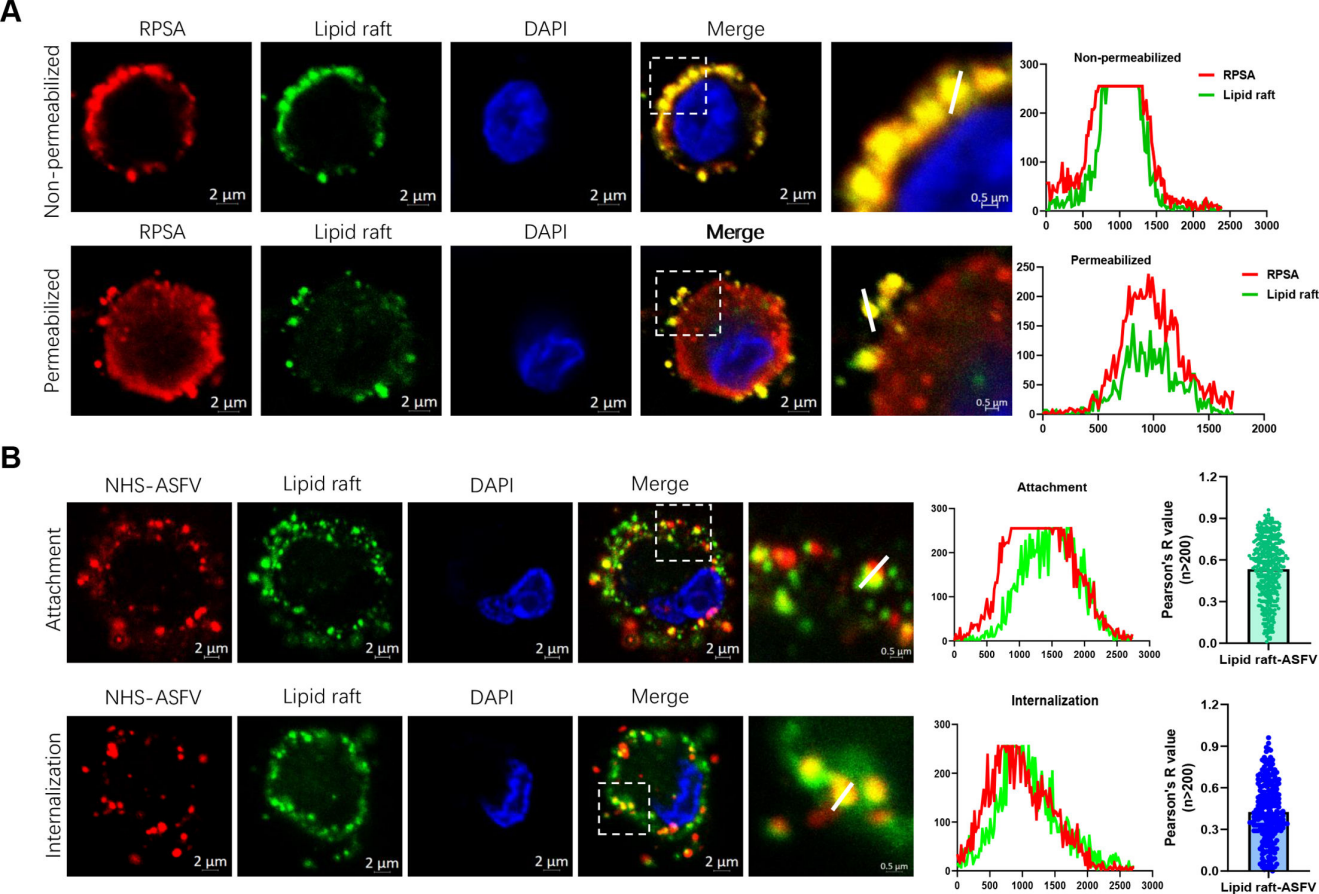
Lipid rafts serve as the platform for ASFV invasion. (**A**) PAMs were fixed with 4% paraformaldehyde in the presence or absence of Triton X-100, followed by immunostaining with antibodies against RPSA and cholera toxin B (CT-B), respectively. Immunofluorescence analysis was conducted to assess the co-localization of RPSA (red) and lipid rafts (green). Scale bar = 2 µm. (**B**) PAMs were infected with NHS-ASFV at 4°C or 37°C for 1 h. Subsequently, cells were washed with 0.2 M glycine (pH = 3) to remove non-internalized viral particles. Immunofluorescence was utilized to evaluate the co-localization of NHS-ASFV (red) and lipid rafts (green). The degree of co-localization was quantified using the Pearson correlation coefficient. Scale bar = 2 µm.

**Fig 8:**
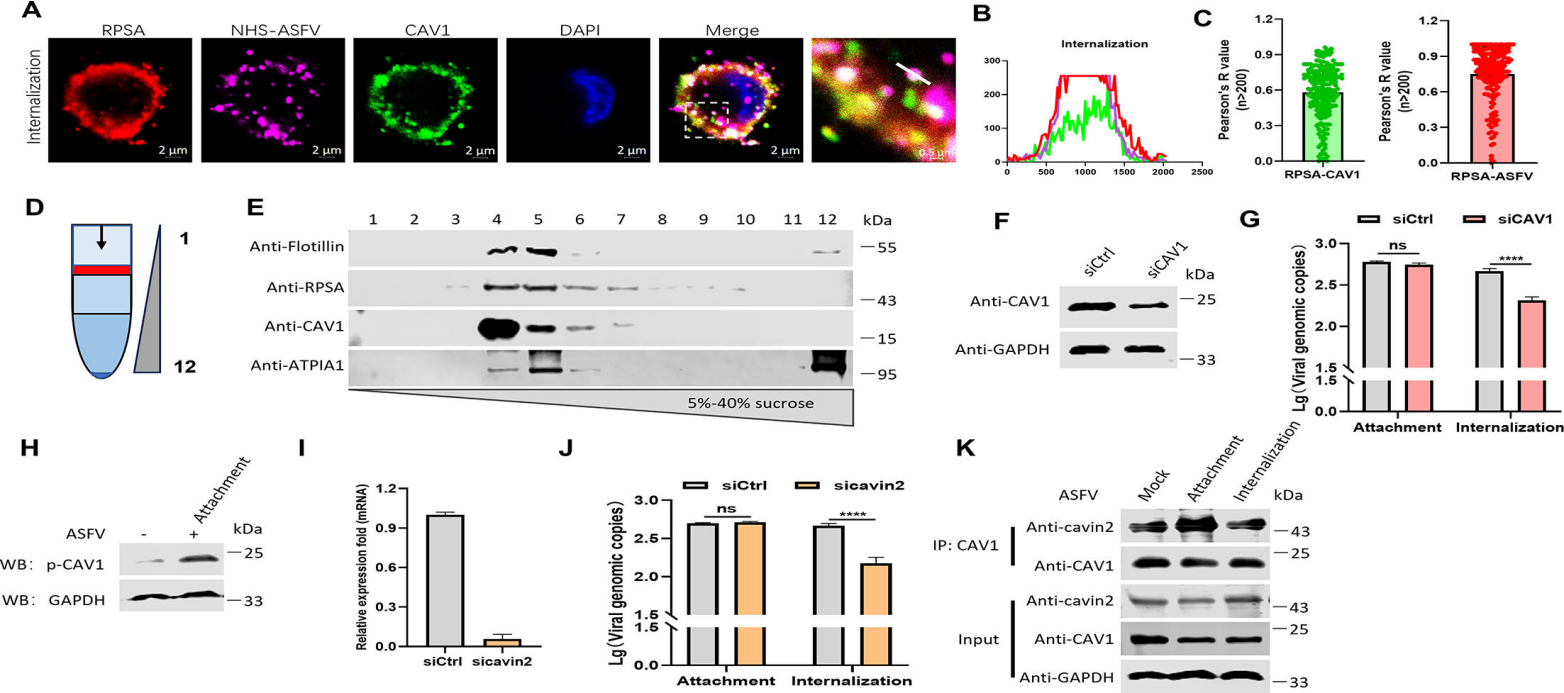
RPSA promotes ASFV entry by collaborating with CAV1-mediated endocytosis via lipid rafts. (**A–C**) PAMs were infected with NHS-ASFV at 37°C for 1 h. Subsequently, the cells were washed with 0.2 M glycine (pH 3) to remove non-internalized virus particles. Immunofluorescence staining was conducted to assess the co-localization of RPSA (red), NHS-ASFV (purple), and CAV1 (green). Scale bar = 2 µm (**A**). Quantitative analysis of the co-localization among RPSA, NHS-ASFV, and lipid rafts (**B**). Over 200 cells were evaluated via fluorescence microscopy, and co-localization was quantified using the Pearson correlation coefficient (**C**). (**D**) Schematic representation of lipid raft isolation by sucrose density gradient ultracentrifugation at the interface between 35% and 5% sucrose layers. (**E**) PAM cell lysates were subjected to sucrose gradient ultracentrifugation to fractionate cellular membranes based on lipid content. Flotillin-1, a lipid raft-associated protein, served as a positive control for lipid raft localization. (**F**) PAMs were transfected with either scrambled control siRNA or siRNAs targeting CAV1 for 24 h. CAV1 protein expression was evaluated by western blot analysis. (**G**) PAMs transfected with siRNA targeting CAV1 were infected with ASFV-WT (MOI = 2) at 4°C or 37°C for 1 h. Following infection, the cells were washed and harvested for quantification of viral genome copies by qPCR. (**H**) PAMs were either mock-infected or infected with ASFV-WT (MOI = 1) at 4°C for 1 h. Phosphorylated CAV1 (p-CAV1) protein levels were assessed by western blot. (**I and J**) PAMs were transfected with scrambled control siRNA or two distinct siRNAs targeting cavin2 for 24 h. Subsequently, cells were infected with ASFV-WT (MOI = 1) at 4°C or 37°C for 1 h. Cavin2 mRNA expression was measured by qPCR (**I**), and intracellular ASFV genomic DNA levels were determined by qPCR (**J**). (**K**) PAMs were mock-infected or infected with ASFV-WT (MOI = 1) at 4°C or 37°C for 1 h. Cell lysates were incubated with anti-CAV1 antibody for 8 h at 4°C, followed by elution of cavin2 using protein A + G Sepharose beads. Cavin2 protein levels were analyzed by western blot. Data are presented as mean ± standard deviation from three independent experiments. Statistical significance is indicated as follows: ****, *P* < 0.0001; ns, not significant (*P* > 0.05).

The caveolae are the most characteristic structures within lipid rafts. Previous reports have shown that lipid rafts are specialized microdomains in the cell membrane that are enriched in cholesterol and sphingomyelin and are characterized by increased membrane rigidity and reduced fluidity, thereby providing an optimal platform for viral adhesion and invasion. We initially observed colocalization between RPSA and lipid rafts (Fig. 7A), as well as colocalization of NHS-labeled ASFV virions with lipid rafts during the processes of attachment and internalization (Fig. 7B). Notably, confocal laser scanning microscopy results revealed the significant colocalization among RPSA, CAV1, and ASFV virions during viral internalization (Fig. 8A through C). Subsequently, lipid raft isolation experiments confirmed the presence of RPSA in the lipid raft fractions (Fig. 8D and E).

Caveolae are primarily composed of CAV1, cavin1, and cavin2 isoforms, with cavin2 playing a critical role in the formation process of endocytic vesicles (31–33). The binding of specific ligands, such as fatty acids, albumin, and certain viruses, to cell membrane receptors triggers the phosphorylation of caveolin-1 at tyrosine-14 (34). Following CAV1 oligomerization, resulting in the formation of a shallow membrane invagination, cavin2 interacts with membrane lipids through its basic disordered region and, together with cavin1, leads to enhanced membrane curvature (32). Subsequently, with the involvement of dynamin and GTP, the neck of the caveolae is “pinched off,” causing it to detach from the plasma membrane and form an independent caveolar vesicle that enters the cell interior (35–37). We also noticed that the expression of CAV1 was knocked down in PAMs cells (Fig. 8F), and the level of internalization of ASFV virions decreased in PAMs transfected with siRNA targeting CAV1 (siCAV1) compared to the siNC-treated group (Fig. 8G). It is well-known that CAV1 undergoes phosphorylation during viral infection, especially viral entry (34). Consistent with these results, we also observed that ASFV infection induced phosphorylation of CAV1 during viral attachment (Fig. 8H). Furthermore, we found that knockdown of cavin2 expression inhibited the internalization of ASFV virions (Fig. 8I and J). Notably, the co-internalization of CAV1 and cavin2 was enhanced during ASFV adhesion, which was conducive to the internalization of ASFV (Fig. 8K). Taken together, our findings indicate that RPSA is involved in CAV1-mediated viral internalization.

### RPSA facilitates ASFV entry by binding with CAV1

**Fig 9:**
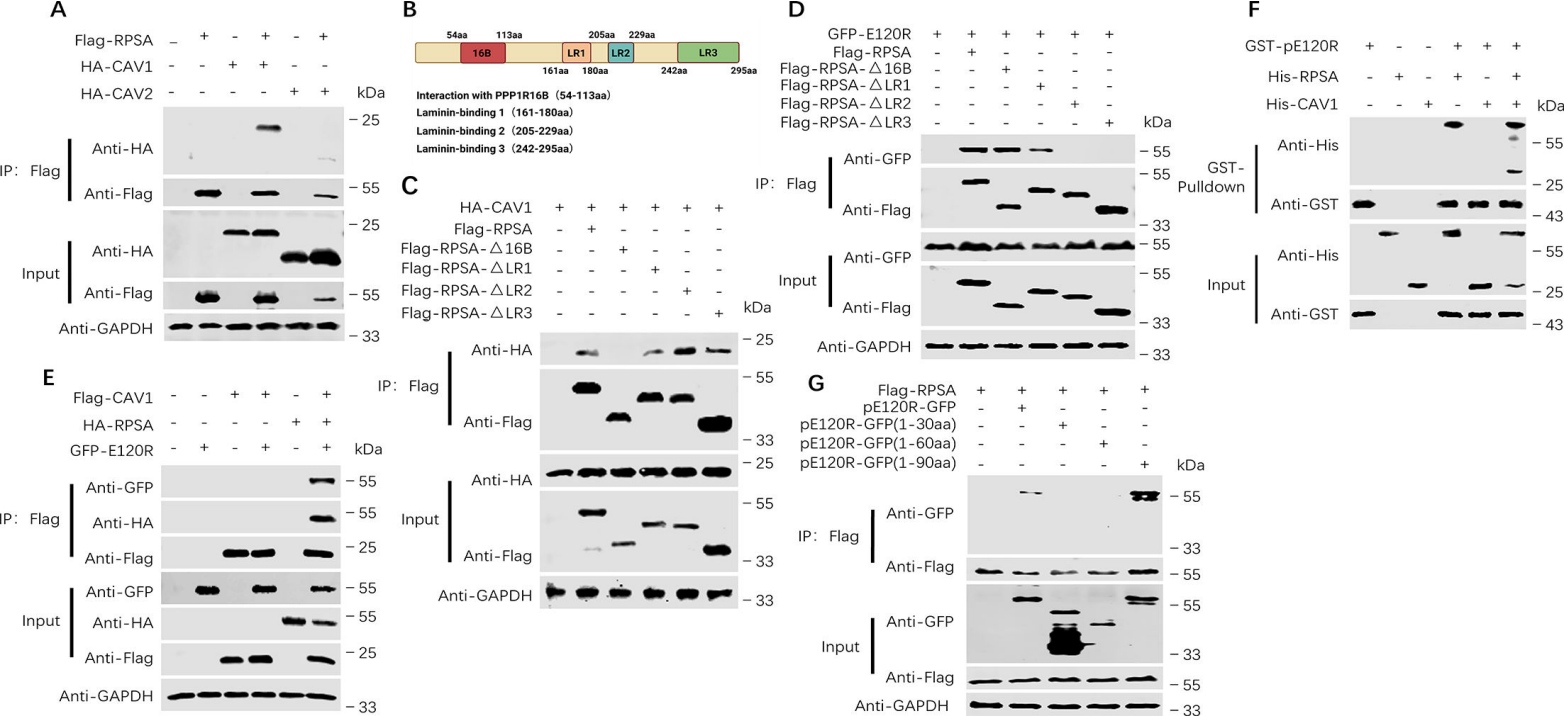
Cooperative role of RPSA and CAV1 in facilitating ASFV entry. (**A**) HEK293T cells were transfected with plasmids encoding Flag-tagged RPSA, HA-tagged CAV1, and HA-tagged CAV2, respectively. Co-IP assays were conducted using an anti-Flag antibody. Both the immunoprecipitates and whole-cell lysates (input) were subjected to immunoblotting with anti-Flag and anti-HA antibodies. (**B**) Diagrammatic representation of RPSA and its corresponding truncation mutants. (**C**) Co-IP analysis assessing the interaction between Flag-RPSA or its truncation mutants and HA-CAV1 in HEK293T cells. (**D**) Co-IP analysis evaluating the interaction between Flag-RPSA or its truncation mutants and GFP-tagged pE120R in HEK293T cells. (**E**) Cell lysates from HEK293T cells overexpressing Flag-CAV1, HA-RPSA, and GFP-pE120R were immunoprecipitated with an anti-Flag antibody. The immunoprecipitates and whole-cell lysates (Input) were immunoblotted using anti-Flag, anti-HA, and anti-GFP antibodies, respectively. (**F**) Recombinant GST-tagged pE120R was incubated with purified His-tagged RPSA (6 × His RPSA) and CAV1 (6 × His-CAV1). The reaction products were analyzed with anti-GST and anti-His antibodies. (**G**) Co-IP analysis of the interaction between GFP-pE120R or its truncation mutants and Flag-RPSA in HEK293T cells. Data are presented as mean ± standard deviation from three independent experiments. Statistical significance is indicated as follows: ****, *P* < 0.0001; ***, *P* < 0.001; *, *P* < 0.05.

To further investigate whether RPSA affects caveola-mediated endocytosis, we first tested the interaction between RPSA and CAV1 by Co-IP assay. Our results demonstrated a specific interaction between RPSA and CAV1 (Fig. 9A). Structurally, RPSA comprises a PPP1R16B-binding domain (16B) and three laminin-binding domains (LR1, LR2, and LR3) (Fig. 9B). To delineate the regions responsible for this interaction, we generated four plasmids expressing RPSA mutants and then assessed their binding to CAV1 via Co-IP. As shown in Fig. 9C, the 16B domain of RPSA mediates its interaction with CAV1. Moreover, we observed that the viral protein pE120R interacted with the LR2 and LR3 domains of RPSA (Fig. 9D). Furthermore, CAV1 was found to associate with pE120R in the presence of RPSA, implying that RPSA may function as a molecular bridge linking CAV1 and pE120R (Fig. 9E and F).

## DISCUSSION

**Fig 10:**
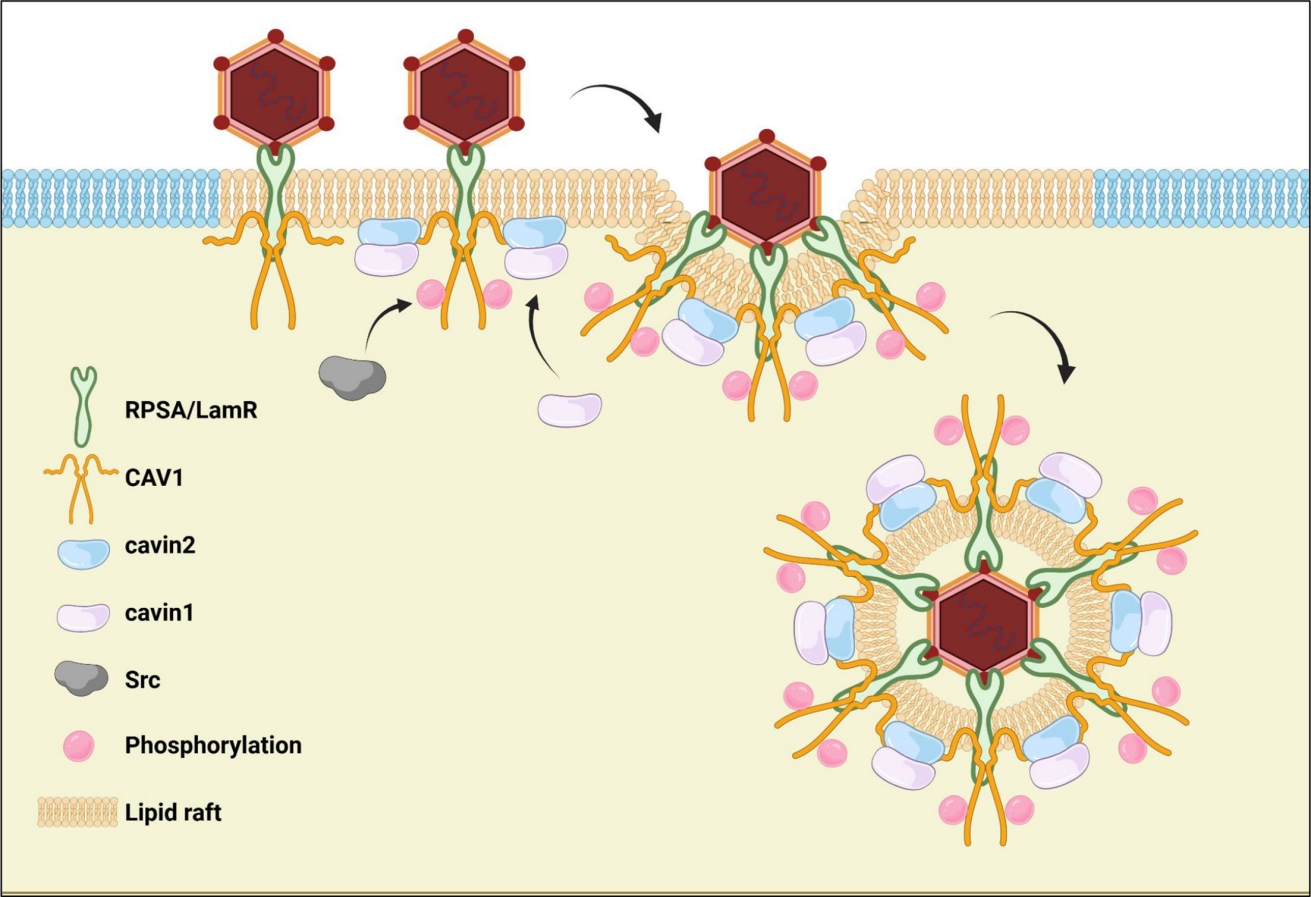
Schematic representation of the role of RPSA in facilitating ASFV virion entry into host cells. ASFV virions gain entry into host cells through the interaction of the host factor RPSA localized within lipid rafts. The viral surface protein pE120R binds to RPSA on the lipid raft, initiating a signal that is subsequently transmitted to caveolin-1 (CAV1) within the same membrane microdomain, thereby establishing the pE120R-RPSA-CAV1 signaling axis. RPSA engages with CAV1 via its 16B domain, which promotes the assembly and aggregation of CAV1-cavin2-cavin1 complexes. This molecular cascade facilitates the endocytosis of ASFV virions during infection.

Although ASF has been recognized for over a century, the precise mechanisms underlying ASFV entry remain incompletely elucidated. In this study, we found that antibody against ASFV pE120R could block ASFV infection in a dose-dependent manner (data not shown), consistent with findings reported previously (7, 38). To further investigate the function of pE120R, we conducted Co-IP/MS to identify host binding partners of pE120R and found that RPSA exhibited the highest interaction scores among the candidate proteins. Moreover, our results indicate that RPSA promotes ASFV entry by mediating viral attachment, which is involved in caveola/lipid raft-mediated endocytosis (CavME) (Fig. 10).

ASFV contains a relatively large, linear double-stranded DNA genome, approximately 170 to 194 kb in length and encodes more than 150 proteins (2, 3). The functions of many ASFV-encoded proteins remain largely uncharacterized. Previous studies have demonstrated that unenveloped ASFV virions, whose major capsid proteins include p72, pE120R, p49, and pH240R, remain infectious. To date, only a limited number of proteins have been confirmed to participate in viral entry, such as p54, pO61R, and p72. Notably, p72 interacts with the host molecule CD1d and facilitates the clathrin-mediated endocytosis of the virus (9). Andrés et al. have demonstrated that pE120R is essential for virus transport from assembly sites to plasma membrane but not for infectivity (39). Recently, Ma et al. also revealed that ASFV-ΔE120R exhibited impaired virion release (40). Additionally, Ma et al. demonstrated that viral particles without pE20R are more susceptible to neutralization by convalescent pig sera, suggesting that pE120R is related to cell invasion. Consistent with Ma’s results, we also found that the antibody against pE120R inhibits ASFV entry (data not shown), suggesting that pE120R is required for ASFV invasion. Therefore, pE120R has a different function involved in viral entry and egress.

RPSA, also known as laminin receptor 1, is ubiquitously expressed across cells and localized throughout the cellular compartments, where it performs multiple functions by regulating viral infections (20–22). Initially, RPSA was identified for its fundamental housekeeping role in the assembly of the 40S ribosomal small subunit. Within the nucleus, RPSA functions as a natural immune sensor (41, 42) by directly recognizing viral DNA or RNA that enter the nucleus. This recognition enhances the recruitment of NF-κB p65 subunit to the promoter regions of pro-inflammatory genes, thereby selectively amplifying NF-κB-mediated inflammatory responses (41). Furthermore, scientists have demonstrated that RPSA activates ERK1/2 signaling following viral entry, which in turn regulates lipid synthesis/transport, as well as upregulates aminopeptidase N (APN) expression. These processes collectively create a favorable environment for viral replication and provide receptors for transmissible gastroenteritis virus (TGEV) and porcine deltacoronavirus, thereby facilitating viral replication (43). In this study, host proteins interacting with pE120R were identified, revealing that RPSA is a prominent candidate. RPSA primarily recognizes and binds to the CDPGYIGSR sequence within the β1 chain of laminin, with the YIGSR pentapeptide being particularly critical for this interaction (44, 45). However, alignment of the amino acid sequence of pE120R protein revealed the absence of this binding motif. We have identified that the amino acid sequence spanning positions 60 to 90 within pE120R is required for interaction with RPSA (Fig. 9G). We used AlphaFold3 to predict the binding amino acid sites between RPSA and pE120R, revealing seven binding sites (Ser-6, Lys-12, Tyr-26, Ile-33, Glu-64, Glu-68, and Asp-71). Most of these binding sites are consistent with our Co-IP results. Therefore, we hypothesize that the amino acid residues Glu-64, Glu-68, and Asp-71 of pE120R may contribute to the interaction between pE120R and RPSA. Subsequently, our results demonstrated that RPSA is involved in ASFV attachment and internalization.

In 2006, membrane rafts were defined as small (10–200 nm) membrane domains enriched in sterols and sphingolipids, which are notable for their heterogeneity and dynamic nature, and functioning to compartmentalize cellular processes (46). Subsequent studies have demonstrated that membrane rafts can be regulated by cholesterol, sphingolipids, and glycophosphatidylinositol (GPI)-anchored proteins within lipid rafts, facilitating the formation of larger platforms exceeding 300 nm in size (47). Previous studies have shown that RPSA predominantly localizes to lipid raft regions on the cell surface, a localization that may be implicated in viral entry (29, 48). Accumulating evidence has identified the 37/67 kDa RPSA as a receptor mediating the entry of various viruses (49, 50). In this study, we found that knockdown of RPSA expression significantly inhibited both the ASFV attachment and internalization in PAMs, whereas overexpression of RPSA enhanced ASFV adsorption and internalization in CV1 cells. Consistent with these results, substantial co-localization of RPSA with lipid rafts was detected. Additionally, the ASFV virions were observed in lipid rafts, showing the co-localization with RPSA. Taken together, our findings provide compelling evidence that RPSA participates in the entry process of ASFV.

ASFV entry has been thought to be governed exclusively by clathrin-mediated endocytosis (CME) and macropinocytosis, forming a seemingly immutable “dual-route” paradigm (51–53). However, Gao et al. recently demonstrated that ASFV can utilize apoptotic bodies for infection and cell-to-cell transmission (54). In this study, we provide the first conclusive evidence that ASFV also exploits a previously unrecognized entry mechanism, caveola-mediated endocytosis (CavME), to facilitate efficient cellular internalization. CAV1, a principal protein associated with lipid rafts and a key component of the CavME pathway, was found to colocalize significantly with ASFV virions and RPSA during viral internalization, as demonstrated by confocal laser scanning microscopy. Moreover, lipid raft purification results also displayed the coexistence of RPSA, CAV1, and lipid rafts within the same membrane fractions. These data suggest that ASFV internalization may be mediated by the CavME pathway.

Caveola-mediated endocytic vesicles are characterized by diameters ranging from 50 to 100 nm (55, 56). However, the average size of ASFV virion is approximately 200 nm, posing a challenge to the conventional understanding of this entry route. Notably, vesicle size in the CavME pathway is modulated by cavin proteins, particularly cavin1 and cavin2 (32). For example, phosphorylation of CAV1 at tyrosine 14 (Tyr14) facilitates the recruitment of cavin1 and cavin2, promoting caveolar vesicle formation (34, 57). The phosphorylation of CAV1 is mainly carried out by Src family kinases (34, 58). SRC, via its N-terminal myristoylation modification, establishes hydrophobic interactions with the lipid raft/caveola microdomains where CAV1 is located, facilitating the phosphorylation of CAV1 at Tyr14. It has been reported that several viruses can enter cells through this pathway (59), including large viruses such as equine herpes virus (EHV) (60) and foot-and-mouth disease virus (FMDV) (61), suggesting that caveola size may not be strictly limited to below 100 nm, thereby providing a theoretical framework for ASFV entry via CavME. Consistent with this hypothesis, we observed a significant increase in CAV1 phosphorylation during ASFV attachment. Furthermore, we also observed an increase in the interaction between cavin2 and CAV1 during ASFV adhesion. Collectively, these results provide evidence for ASFV entry through the caveolin-mediated endocytosis pathway.

In summary, our study demonstrated that the capsid protein pE120R on the ASFV virions binds to the host membrane raft component RPSA. This interaction subsequently facilitates the recruitment of CAV1, resulting in the formation of the pE120R-RPSA-CAV1 complex, which mediates the endocytosis of ASFV virions during ASFV infection. Our findings enhance the understanding of RPSA’s functional role and offer novel insights into the mechanism of ASFV entry, thereby providing a theoretical foundation for the development of antiviral therapeutic strategies

## MATERIALS AND METHODS

### Cell and virus

PAMs were prepared from lung lavage of 4-week-old specific-pathogen-free (SPF) piglets, as previously described and maintained in RPMI 1640 supplemented with 10% heat-inactivated fetal bovine serum (FBS: Gibco, Carlsbad, CA), 100 U/mL penicillin, and 100 mg/mL streptomycin. HEK293T cells and CV-1 cells obtained from the American Type Culture Collection (ATCC) were cultured in Dulbecco’s modified Eagle’s medium (DMEM) supplemented with 10% FBS and penicillin-streptomycin. All the cells were maintained at 37°C with 5% CO_2_. The ASFV isolates HLJ/2018 (GenBank accession number: MK333180.1) were isolated from the pigs, as previously described. A recombinant ASFV expressing Gaussia luciferase (Gluc) and GFP (rASFV-Gluc-GFP) was generated from ASFV HLJ/18 isolate and maintained in our laboratory. In brief, two reporter expression boxes expressing Gluc and GFP were inserted into the K145R gene site of ASFV using CRISPR/Cas9 gene editing and homologous recombination techniques. There was no difference in growth kinetics between the parent virus and the recombinant virus.

### Plasmids

The RPSA overexpressing plasmid was constructed by inserting the cDNA of RPSA into the mammalian expression vector pCAGGS with a Flag tag at the N-terminus. The overexpressing plasmids CAV1 and CAV2 were constructed by inserting the cDNA of CAV1 and CAV2 into pCAGGS with a HA tag at the N-terminus. The E120R gene of ASFV was cloned into pCAGGS with a GFP tag at the C-terminus.

### Antibodies and reagents

Anti-pE120R, anti-p72, and anti-p30 antibodies were obtained from our laboratory, and other antibodies were purchased commercially: rabbit anti-RPSA antibody (14533-1-AP, Proteintech), mouse anti-RPSA antibody (67324-1-Ig, Proteintech), rabbit anti-HA and anti-Flag antibodies (3724S and 14793S, CST), mouse anti-His and anti-GST antibodies (66005-1-Ig and 66001-2-Ig, Proteintech), rabbit anti-CAV1 antibody (ab17052, Abcam), rabbit anti-Flotillin antibody (67968-1-lg, Proteintech), rabbit anti-GAPDH, anti-ATP1A1, and anti-GFP antibodies (10494-1-AP, 14418-1-AP and 50430-2-AP, Proteintech); IRDye 800CW goat anti-mouse IgG (H + L) and IRDye 800CW goat anti-rabbit IgG (H + L) (926-32210 and 926-32211, LI-COR); Mem-PER Plus Membrane Protein Extraction Kit (89,842, Thermo Fisher); Vybrant Alexa Fluor 488 Lipid Raft Labeling Kit (V34403, ThermoFisher Scientific); and CPZ (31,679, Sigma-Aldrich), EIPA (HY-101840, MCE), and nystatin (N9150, Sigma-Aldrich).

### Co-immunoprecipitation (Co-IP)

Cells were collected and lysed in 1% NP-40 lysis buffer with protease inhibitors. Cellular debris was removed by centrifugation at 12,000 rpm for 20 min at 4°C. The lysates were immunoprecipitated with antibodies and subsequently adsorbed onto Protein A/G Agarose (SC-2003, Santa Cruz). The cell lysates and immunoprecipitants were resolved by 12% SDS-PAGE and then transferred to polyvinylidene difluoride (PVDF) membranes (ISEQ00010, Merck Millipore) for immunoblot analysis.

### Western blot

The plasmid-transfected cells were washed twice with cold PBS and lysed with 1% NP-40 lysis buffer with protease inhibitors. Cell lysates were centrifuged at 12,000 rpm at 4°C for 20 min. The supernatants were then mixed with protein sample loading buffer and boiled for 10 min. The samples were loaded onto 12% SDS-PAGE and separated by electrophoresis. Proteins were transferred to a PVDF membrane. After blocking with 5% BSA, the membrane was incubated sequentially with the indicated primary and secondary antibodies and then detected using the Odyssey imaging system (LI-COR, USA).

### Mass spectrometry

The ASFV pE120R-binding proteins in the immunoprecipitants were resolved by 12% SDS-PAGE, and the silver-stained gel was cut and processed for liquid chromatography-tandem mass spectrometry (LC-MS/MS) (probability-based protein identification by searching sequence databases using mass spectrometry data). The MS/MS signals were then processed against the National Center for Biotechnology Information (NCBI) protein database by using the Mascot Server (Matrix Science). High-confidence peptides with a prerequisite of a minimum of two peptides leading to the identification of proteins were selected and listed in Table S1. The protein “score” displays the standard score: the cumulative protein score based on summing the ion scores of the unique peptides. A higher score indicates higher confidence in identification.

### Immunofluorescence assays (IFA)

Cells grown on a poly-L-lysine-coated glass-bottom cell culture dish were fixed in 4% paraformaldehyde for 30 min and washed one time with 1× PBS (pH = 7.4). The cells were then permeabilized in 0.2% Triton X-100 in 1× PBS and blocked with 5% BSA in 1× PBS for 1 h. Then, the cells were incubated with anti-Flag and anti-HA antibodies for 1 h and then stained with indicated secondary antibodies for 1 h. Nuclear DNA staining was performed with DAPI (Sigma). Samples were visualized with a Zeiss LSM-800 laser scanning fluorescence microscope (Carl Zeiss AG, Oberkochen, Germany). For the ASFV attachment assay, non-permeabilized cells were used and detected with IFA. Then, cells were incubated with mouse anti-pE120R antibody. For assessment of the colocalization of pE120R and RPSA, PAMs were infected for 24 h, and the cells were incubated with mouse anti-pE120R antibody and rabbit anti-RPSA antibody. Colocalization analyses were performed using the Pearson correlation coefficient showing an actual overlap of the signals, which is considered to represent the true degree of two proteins’ colocalization.

### GST-pulldown assay

GST-PE120R, His-RPSA, and His-CAV1 proteins were produced by *E. coli* BL21 (DE3). GST-pE120R was incubated with GST-tag purification resin for 4 h at 4°C. The resin was washed five times with precooled PBS. Then, GST-p30 was prepared by re-suspending the resin with PBS. The GST-pE120R (40 μL) was separately incubated with His-RPSA and His-CAV1 (40 μL) for 6 h at 4°C. After incubation, the gel was washed three times with cold PBS, followed by western blot.

### Quantitative PCR (qPCR)

PAMs were collected, and total RNAs were extracted using TRIzol reagent (Invitrogen, Carlsbad, CA, USA). The reverse transcription cDNAs were prepared by using PrimeScript RT Reagent Kit (Takara, Kusatsu, Shiga, Japan) according to the manufacturer’s instructions. To detect RPSA mRNA expression, the cDNAs were amplified using a QuantStudio 5 system (Applied Biosystems, Sunnyvale, CA, USA) with SYBR Premix ExTaq II (Takara, Kusatsu, Shiga, Japan). The relative mRNA levels of these genes were evaluated by the 2−ΔΔCT method using hypoxanthine phosphoribosyl transferase (HPRT) mRNA as an endogenous control. The primers for RT-qPCR are listed in Table S2. For ASFV genomic DNA copy detection, ASFV genomic DNA was extracted from cells using a Qiagen DNA Mini Kit (Qiagen, Germany). The qPCR was carried out on a QuantStudio 5 system according to the OIE-recommended procedure.

### ASFV infection and RNA interference

PAMs were seeded into 24-well plates at 2 × 10^5^ cells per well and infected with ASFV (MOI = 1) for 2, 12, and 24 h, respectively. At different time points, the cells were harvested. All siRNAs and siRNA controls (siCtrl) were designed and synthesized by Sangon (Shanghai, China) and listed in Table S2. In knockdown experiments, PAMs were transfected with siRNA negative controls or siRNAs as indicated at a final concentration of 10 nM using Lipofectamine RNAiMAX, according to the manufacturer’s instructions. Twenty-four hours after transfection, cells were infected with ASFV (MOI = 1) for 24 h. The total RNA and DNA of cells were extracted for qPCR.

### Luciferase reporter gene assay

Gaussian luciferase activities were measured with a Luciferase Reporter Assay System (PierceTM Gaussia Luciferase Flash Assay Kit) according to the manufacturer’s instructions.

### Antibody blocking assay

PAMs were seeded in 48-well plates overnight and then incubated with different concentrations of anti-RPSA antibody for 1 h at 4°C. After being washed with fresh media, the cells were infected with rASFV-Gluc-GFP (MOI = 1) for 24 h. As a control, PAMs were pretreated in parallel with rabbit IgG (Proteintech). The cells were harvested for total DNA extraction and ASFV genomic DNA copy detection.

### Flow cytometry

CV-1 cells were transfected with pCAGGS-Flag-RPSA or pCAGGS for 24 h and infected ASFV for 24 h, then collected in a 1.5 ml tube. The cells were washed three times with PBS and fixed with 4% paraformaldehyde at room temperature for 30 min. The cells were analyzed using a FC-500 flow cytometer (Beckman Coulter).

### Membrane protein extraction

CV-1 cells were transfected with pCAGGS-Flag-RPSA for 24 h. Membrane proteins were isolated from 3 × 10^8^ cells using the Mem-PER Plus Membrane Protein Extraction Kit (Thermo Fisher Scientific) following the manufacturer’s instructions. The plasma membrane proteins were analyzed by western blotting.

### Isolation and purification of lipid rafts

The cells were washed three times in ice-cold TNE buffer (25 mM Tris HCl, 150 mM NaCl, 5 mM EDTA, pH 6.7), then lysed on ice with 1 ml of lysis buffer (1% Triton X-100 in TNE, supplemented with a complete protease inhibitor cocktail) with shaking for 30 min using 107 cells. Subsequently, the lysate was passed 20 times through an 18-gauge needle on a 1 mL syringe, and then mixed with 2 mL of ice-cold 60% sucrose. The mixture was placed at the bottom of a Beckman SW41 ultracentrifuge tube (Beckman, Munich, Germany), overlay it with 5 mL of ice-cold 35% sucrose in TNE and 3 mL of ice-cold 5% sucrose in TNE, and then centrifuge at 200,000 × *g* for 20 h at 4°C. Twelve fractions were collected from the top to the bottom of the tube.

### Establishment of stable cell lines overexpressing RPSA

The cDNA of the porcine RPSA sequence (NCBI Reference Sequence: NM_001037146.2) was cloned into a pLVX-IRES-Puro vector to generate lentivirus via the psPAX2-pMD2G system in HEK293 T cells. CV-1 cells were transduced with packaged lentivirus expressing RPSA and cultured in a medium supplemented with puromycin (Gibco) for selection. The surviving cell clone was isolated, propagated, and examined for stable expression of RPSA by western blot.

### Virus binding and internalization assay

PAMs cells were infected with ASFV (MOI = 2) and incubated at 4°C for 1 h. The unbound virus was removed by two washes with 1× PBS. The dishes were overlaid with a complete medium and shifted to 37°C for 2 h. The infected cells were treated with 0.2 M glycine (pH = 3) for 2 min at room temperature to inactivate extracellular viruses and then washed twice with 1× PBS. Cells were harvested for total DNA extraction and ASFV genomic copy detection. As a control for each experiment, adsorbed viruses were removed using acid glycine washes and are shown in the experiments as 4°C + Gly.

### Preparation of NHS-labeled ASFV (NHS-ASFV) for binding and internalization

To prepare NHS-ASFV, purified ASFV HLJ/2018 particles were incubated with 100 nM NHS (Thermo) in 1× PBS for 1 h at room temperature and then centrifuged at 5,000 × *g* for 1 h at 4°C to remove the supernatant through Amicon Ultra (UFC9100, Merck Millipore). The sediment was washed with 1× PBS twice and resuspended in 1× PBS, and the resulting preparation was named NHS-ASFV. PAMs were infected with NHS-ASFV at 4°C for 1 h, washed to remove the free NHS-ASFV, and then transferred to 37°C for up to 1 h to allow incorporation of NHS-ASFV. The cells were incubated with or without anti-p72 antibody and observed using high-magnification confocal microscopy for NHS-ASFV binding and internalization. To quantify the binding and internalization of NHS-ASFV, we used WGA-labeled cell membranes (gray) as a reference, where colocalized (red) with the cell membrane was considered the binding virus and internal (red) signal represented the internalized virus. More than 200 cells were counted per sample, and the percentage of cells that internalized NHS-ASFV positive was calculated.

### Nystatin inhibitor assay

PAMs were treated with different concentrations (10, 20, and 30 μM) of nystatin for 30 min, then infected with ASFV HLJ/2018 (MOI = 1) for 1 h at 4°C. Next, the cells were washed to remove any unbound ASFV and incubated at 37°C for 1 h. DMSO was used as a control. The total DNA of cells was extracted for the detection of ASFV genomic DNA copies.

### Statistical analysis

In the present study, GraphPad Prism 8.0 was used for statistical calculations and data plotting. Differences between two independent samples were evaluated by two-tailed Student’s *t*-tests. Differences between multiple samples were analyzed by one-way analysis of variance (ANOVA). The data are presented as mean ± SD. We considered *P* < 0.05 to be statistically significant. Significance values were set as follows: ns (not significant), *P* > 0.05; ****, *P* < 0.0001; ***, *P* < 0.001; **, *P* < 0.01; *, *P* < 0.05.

## Data Availability

All relevant data are within the article and its supplemental material.

## SUPPLEMENTAL MATERIAL

Supplemental material jvi.00131-26-s0001.docx Tables S1 and S2.

ASM does not own the copyrights to Supplemental Material that may be linked to, or accessed through, an article. The authors have granted ASM a non-exclusive, world-wide license to publish the Supplemental Material files. Please contact the corresponding author directly for reuse.
