# Supplementary material for: RPSA enhances African swine fever virus entry via caveola-mediated endocytic pathway
Source: J Virol. 2026 May 11;100(6):e00131-26. doi: 10.1128/jvi.00131-26 (PMC13289060; doi:10.1128/jvi.00131-26)
Supplement: Supplemental material — Tables S1 and S2. [file jvi.00131-26-s0001.docx]

**Supplementary Information**

RPSA enhances African Swine Fever Virus entry via Caveolae-Mediated Endocytic Pathway

Chuanxia Liu ^a^^, #^, Tingting Li ^a, #^, Yibing Wang ^a^, Fei Zhao ^a^, Li Huang ^a, b^, Li Huang ^a, b, *^, Changjiang Weng ^a, b,^

^a^ Division of Fundamental Immunology, National African Swine Fever Para-reference Laboratory, State Key Laboratory for Animal Disease Control and Prevention, Harbin Veterinary Research Institute, Chinese Academy of Agricultural Sciences (CAAS), Harbin, 150069, China.

^b^ Heilongjiang Provincial Key Laboratory of Veterinary Immunology, Harbin, 150069, China.

| **Number** | **Protein description** | **Mass** | | **Score** | **Matches** | **Coverage** |
| --- | --- | --- | --- | --- | --- | --- |
| 1 | RPSA | | 38864 | 898 | 104(43) | 28% |
| 2 | Vimentin | | 66827 | 207 | 11(5) | 22% |
| 3 | TPM3 | | 66307 | 176 | 19(10) | 24% |
| 4 | GNB2L1 | | 57390 | 170 | 9(5) | 41% |
| 5 | Arp2 | | 53692 | 161 | 20(8) | 37% |
| 6 | EF-G | | 53692 | 133 | 11(5) | 44% |
| 7 | SLC39A11 | | 36717 | 132 | 13(8) | 23% |
| 8 | ETF-α | | 33678 | 126 | 21(16) | 25% |
| 9 | Gelsolin | | 36605 | 115 | 20(12) | 20% |
| 10 | PRSS1 | | 30131 | 113 | 12(9) | 26% |
| 11 | Hsp70 | | 29864 | 109 | 14(7) | 5% |
| 12 | Myosin IC | | 29019 | 105 | 10(3) | 5% |
| 13 | HSPA5 | | 26386 | 103 | 4(3) | 19% |
| 14 | hnRNP U | | 30148 | 88 | 8(4) | 14% |
| 15 | ACAA1 | | 23139 | 83 | 14(8) | 26% |
| 16 | CPS1 | | 17968 | 76 | 8(6) | 5% |
| 17 | eIF3A | | 11686 | 71 | 4(3) | 11% |
| 18 | Rab3 | | 16434 | 68 | 7(4) | 20% |
| 19 | TMEM203 | | 23139 | 65 | 4(2) | 5% |

**Supplementary Table 1. Identification of pE120R binding partners by mass spectrometry.**

**Supplementary Table 2. The sequence of siRNA and primers used in this study.**

| **siRNA** | **Sequence (5'-3')** |
| --- | --- |
| si-RPSA-213 | AGCCGAUGUCAGUGUCAUATT |
| si-RPSA-428 | CUACCAUUGCUCUGUGUAATT |
| si-CAV1-5 | CAGAGGAAAUGAACGAGAATT |
| si-CAV1-90 | CGACGUUGUCAAGAUUGAUTT |
| si-CAV1-179 | CCUUCACUGUGACGAAGUATT |
| si-cavin2-1119 | GGCUAUUGUGGAAGAUGAATT |
| si-cavin2-143 | GGGACAACUCGCAGGUGAATT |
| si-cavin2-549 | GGAAGAACUUGCAGAUGAATT |
| si-Ctrl | UUCUCCGAACGUGUCACGUTT |
| **Primer** | **Sequence (5'-3')** |
| **qPCR** | |
| ASFV-p72-F | CTGCTCATGGTATCAATCTTATCGA |
| ASFV-p72-R | GATACCACAAGATCAGCCGT |
| HPRT-F | GCCGAGGATTTGGAAAAGG |
| HPRT-R | GCACACAGAGGGCTACGATG |
| ASFV-probe | FAM-CCACGGGAGGAATACCAACCCAGTG-TAMRA |
| Forward-RPSA | AGATTGGTCCGAAGGTGTGC |
| Reverse-RPSA | GGTAGGCTGAGCACTCCAAT |
| Forward-CAV1 | GGGCAACATCTACAAGCCCA |
| Reverse-CAV1 | GATCGCGGTTGACCAAGTCT |
| Forward-cavin2 | CAGGGAAAAGCTCCCCCTTC |
| Reverse-cavin2 | GCTCTCCTCATGGTCGTTGG |
| **Recombinant expression** | |
| pCAGGS-HA-RPSA-F | TTTGGCAAAGAATTCATGTACCCATACGACGTCCCAGACTACGCTATGTCCGGAGCCCTCGATGTCC |
| pCAGGS-HA-RPSA-R | GGGAAAAAGATCTGCTAGCTCGAGTTAAGACCACTCAGTGGTTGTTCC |
| pCAGGS-Flag-RPSA-F | ATGACGACGATAAGGAATTCATGTCCGGAGCCCTCGATGTCCTGC |
| pCAGGS-Flag-RPSA-R | AAAAAGATCTGCTAGCTCGAGTTAAGACCACTCAGTGGTTGTTCCT |
| pCAGGSA-E120R-(1-30)-GFP-F1 | GCAAAGCCACCATGGAATTCATGGCAGATTTTAATTCTCCAAT |
| pCAGGS-E120R-(1-30)-GFP-R1 | AGCTCCTCGCCCTTGCTCACGTCGGCATTTTCATCGTATTCTAGAGAACCTATA |
| pCAGGS-E120R-(1-30)-GFP-F2 | ATGAAAATGCCGACGTGAGCAAGGGCGAGGAGCTGTTCACCGGGGTGGTGC |
| pCAGGS-E120R-(1-30)-GFP-R2 | GAAAAAGATCTGCTAGCTCGAGTTACTTGTACAGCTCGTCCATGCCGAG |
| pCAGGSA-E120R-(1-30)-GFP-F1 | GCAAAGCCACCATGGAATTCATGGCAGATTTTAATTCTCCAAT |
| pCAGGS-E120R-(1-60)-GFP-R1 | CTCGCCCTTGCTCACTTGTGAATACAGGGAAGTTGATGTGGTAGGGTCAT |
| pCAGGS-E120R-(1-60)-GFP-F2 | ACTTCCCTGTATTCACAAGTGAGCAAGGGCGAGGAGCTGTTCACCGGGGT |
| pCAGGS-E120R-(1-90)-GFP-R | TCCTCGCCCTTGCTCACCTCGAGCTCATCGGGGACCAGTGAAGTAA |
| pCAGGS-E120R-(1-90)-GFP-F2 | CTGGTCCCCGATGAGCTCGAGGTGAGCAAGGGCGAGGAGCTGTTCA |
| pCAGGS-Flag-E120R-GFP-F | GCTCGCATAAATCGAAGCTCGAGGTGAGCAAGGGCGAGGAGCTGTT |
| pCAGGS-Flag-E120R-GFP-R | GAGGGAAAAAGATCTGCTAGCTCGAGTTACTTGTACAGCTCGTCCATGC |
| pCAGGS-HA-CAV1-F | GACGTCCCAGACTACGCTGAATTCATGTCGGGGGGCAAATACGTA |
| pCAGGS-HA-CAV1-R | AAAAGATCTGCTAGCTCGAGTTATATTTCTTTCTGCATGTTG |
| pCAGGS-HA-CAV2-F | ACGACGTCCCAGACTACGCTGAATTCATGGGGCTGGAGACTGAGAA |
| pCAGGS-HA-CAV2-R | GAAAAAGATCTGCTAGCTCGAGTCAGTCATGGCTCAGTTGCAGGCT |
| pCAGGS-Flag-RPSA-△16B-R1 | GCTCCCGGAAGGCTGCGGTTCTCTTCAGATTTATGATGTAGA |
| pCAGGS-Flag-RPSA-△16B-F2 | ATCATAAATCTGAAGAGAACCGCAGCCTTCCGGGAGCCAAG |
| pCAGGS-Flag-RPSA-△16B-R2 | GAAAAAGATCTGCTAGCTCGAGTTAAGACCACTCAGTGGTTGTTCC |
| pCAGGS-Flag-RPSA-△LR1-F2 | CTGCGTTATGTGGACATTGCCATCGAAGTTCTGCGCATGCGTG |
| pCAGGS-Flag-RPSA-△LR1-R1 | GGGAGATGGTGCCACGCATGCGCAGAACTTCGATGGCAATGTCCACA |
| pCAGGS-Flag-RPSA-△LR1-R2 | GAAAAAGATCTGCTAGCTCGAGTTAAGACCACTCAGTGGTTGTTCC |
| pCAGGS-Flag-RPSA-△LR2-F | TGACCTCTACTTCTACAGAGAATGGACTGCGCCAGCT |
| pCAGGS-Flag-RPSA-△LR2-R | CGCAGTCCATTCTCTGTAGAAGTAGAGGTCAGGCATGA |
| pCAGGS-Flag-RPSA-△LR3-R | AAAAAGATCTGCTAGCTCGAGTTATTGAGTGGCAGTGAACTCA |
| **Overexpression of RPSA in non-permissive cells** | |
| pLVX-IRES-RPSA-HA-F | ATCTATTTCCGGTGAATTCGCCACCATGTCCGGAGCCCTCGATGTC |
| pLVX-IRES-RPSA-HA-R | CGGGATCCGCGGCCGCtcaAGCGTAGTCTGGGACGTCGTATGGGTAAGACCACTCAGTGGTTGTTC |
